# Male‐to‐female gender dysphoria: Gender‐specific differences in resting‐state networks

**DOI:** 10.1002/brb3.691

**Published:** 2017-04-05

**Authors:** Benjamin Clemens, Jessica Junger, Katharina Pauly, Josef Neulen, Christiane Neuschaefer‐Rube, Dirk Frölich, Gianluca Mingoia, Birgit Derntl, Ute Habel

**Affiliations:** ^1^ Department of Psychiatry, Psychotherapy and PsychosomaticsMedical SchoolRWTH Aachen UniversityAachenGermany; ^2^ JARA‐Translational Brain MedicineJülichGermany; ^3^ Department of Gynecological Endocrinology and Reproductive MedicineMedical SchoolRWTH Aachen UniversityAachenGermany; ^4^ Department of Phoniatrics, Pedaudiology and Communication DisordersMedical SchoolRWTH Aachen UniversityAachenGermany; ^5^ Interdisciplinary Center for Clinical Research (IZKF)RWTH Aachen UniversityAachenGermany; ^6^ Department of Psychiatry and PsychotherapyUniversity of TübingenTübingenGermany; ^7^ Werner Reichardt Center for Integrative Neuroscience (CIN)University of TübingenTübingenGermany; ^8^ LEAD Graduate School and Research NetworkTübingenGermany

**Keywords:** functional network connectivity, gender differences, gender dysphoria, resting‐state, transsexualism

## Abstract

**Introduction:**

Recent research found gender‐related differences in resting‐state functional connectivity (rs‐FC) measured by functional magnetic resonance imaging (fMRI). To the best of our knowledge, there are no studies examining the differences in rs‐FC between men, women, and individuals who report a discrepancy between their anatomical sex and their gender identity, i.e. gender dysphoria (GD).

**Methods:**

To address this important issue, we present the first fMRI study systematically investigating the differences in typical resting‐state networks (RSNs) and hormonal treatment effects in 26 male‐to‐female GD individuals (MtFs) compared with 19 men and 20 women.

**Results:**

Differences between male and female control groups were found only in the auditory RSN, whereas differences between both control groups and MtFs were found in the auditory and fronto‐parietal RSNs, including both primary sensory areas (e.g. calcarine gyrus) and higher order cognitive areas such as the middle and posterior cingulate and dorsomedial prefrontal cortex. Overall, differences in MtFs compared with men and women were more pronounced before cross‐sex hormonal treatment. Interestingly, rs‐FC between MtFs and women did not differ significantly after treatment. When comparing hormonally untreated and treated MtFs, we found differences in connectivity of the calcarine gyrus and thalamus in the context of the auditory network, as well as the inferior frontal gyrus in context of the fronto‐parietal network.

**Conclusion:**

Our results provide first evidence that MtFs exhibit patterns of rs‐FC which are different from both their assigned and their aspired gender, indicating an intermediate position between the two sexes. We suggest that the present study constitutes a starting point for future research designed to clarify whether the brains of individuals with GD are more similar to their assigned or their aspired gender.

## 1 Introduction

“It is fatal to be a man or woman pure and simple; one must be woman‐manly or man‐womanly. … Some marriage of opposites has to be consummated.” (Virginia Woolf, A Room of One's Own, 1929). As this quote by one of the foremost modernists of the twentieth century, English writer Virginia Woolf, indicates, the question of (assigned or aspired) gender is a rather complex one. Interestingly, some people do not identify with the gender they were assigned at birth, but because of the psychological, hormonal, behavioral, or genetic factors rather identify with a gender different from the one they were assigned at birth. The official term describing the distress resulting from a discrepancy between anatomical sex and gender identity is gender dysphoria (GD) (American Psychiatric Association, 2013). It represents a controversial diagnosis (Shechner, 2010) and can result in the need of clinical treatment to support gender transition. Since 1980, the APA's Diagnostic and Statistical Manual (DSM) officially lists GD as a psychiatric diagnosis, which is mostly treated by applying psychotherapy, hormone replacement therapy, and sex reassignment surgery, either separately or in conjunction.

Although the etiopathogenesis is insufficiently resolved (Medras & Jozkow, 2010), recent findings strongly point toward neurobiological influences (Swaab & Garcia‐Falgueras, 2009). Post‐mortem studies found a similarity in the bed nucleus of the stria terminalis (BSTc) between GD individuals and those of their desired gender (Garcia‐Falgueras & Swaab, 2008; Kruijver et al., 2000). However, *in vivo* results of studies using structural magnetic resonance imaging (MRI) are more diverse, ranging from no structural differences between men and male‐to‐female GD individuals (MtFs) (Savic & Arver, 2011), differences between MtFs and men and women (Luders et al., 2009), to an intermediate position for hormonally untreated MtFs between male and female brains (Rametti et al., 2011). Female‐like structures in female‐to‐male GD individuals before hormonal treatment (FtMs) (Rametti et al., 2010) and structural changes through hormonal treatment (Rametti et al., 2012) have also been observed. Functional MRI (fMRI) studies provide a similarly complex pattern with activation similarities between GD individuals and their aspired gender (Carrillo et al., 2010; Gizewski et al., 2009; Schoning et al., 2010; Sommer et al., 2008; Ye et al., 2011) in several tasks sensitive to sex and/or gender differences in neural activity (Lykins, Meana, & Strauss, 2008; Semrud‐Clikeman, Fine, Bledsoe, & Zhu, 2012; Thomsen et al., 2000). However, the number and the reported sample sizes of fMRI studies investigating such differences is small, and therefore drawing definite conclusions about whether the brain activity of GD individuals is more similar to their aspired or their assigned gender is difficult. Furthermore, such task‐related fMRI studies might be biased or confounded by the fact that they mostly employ tasks that are specifically designed to elicit sex and/or gender differences in neural activity, i.e. men and women are supposed to react differently because of the nature of the task presented. A more unbiased approach to study brain activity in GD and eliminate the potential confounding influence of gender‐specific tasks is provided by resting‐state functional connectivity analyses.

In recent years, functional connectivity (FC) fMRI approaches found several brain regions whose spontaneous low‐frequency fluctuations (<0.1 Hz) of the blood oxygen level‐dependent (BOLD) signal registered during resting‐state (rs) correlate with each other. Those regions are believed to be functionally connected (Biswal, Van Kylen, & Hyde, 1997; Biswal et al., 2010; Greicius, Krasnow, Reiss, & Menon, 2003; van den Heuvel & Hulshoff Pol, 2010). Based on their functional and/or anatomical overlap with well‐known functional networks, distinct resting‐state functional networks (RSN) have been identified (Beckmann & Smith, 2005; Damoiseaux et al., 2006; Rosazza & Minati, 2011; Seeley et al., 2007; Smith et al., 2009, 2013). It is believed that these RSN reflect the brain's function beyond explicit tasks and represent the intrinsic functional architecture of the human brain (Sadaghiani & Kleinschmidt, 2013; Smith et al., 2009).

Concerning the influence of gender on the RSN, a few studies have been conducted with mixed results: there is evidence for gender differences in resting‐state FC (rs‐FC) in specific brain regions such as the amygdala (Dai et al., 2012; Kogler et al., 2016), insula (Li, Qin, Jiang, Zhang, & Yu, 2012) and within the sensorimotor network (Allen et al., 2011). In addition, men showed stronger connectivity in parieto‐temporal regions, and within cognitive and sensory networks. Women revealed stronger connectivity in fronto‐temporo‐cerebellar regions, and within attention and memory‐related networks (Filippi et al., 2012). However, Weissman‐Fogel, Moayedi, Taylor, Pope, and Davis (2010) found no gender‐specific differences in cognitive or default mode networks, possibly because of a smaller sample size. Even less is known about differences in the RSN between individuals with GD and men and women. To the best of our knowledge, there is only one single‐case study comparing rs‐FC of one untreated FtM individual with samples of men and women, using seed‐voxel and atlas‐based region‐of‐interest approaches (Santarnecchi, Vatti, Dettore, & Rossi, 2012). In contrast to the aforementioned task‐related functional studies, this FtM revealed a stronger similarity to his biological as compared with his aspired gender in several predefined brain regions sensitive to gender dimorphism. This discrepancy in the results between task‐related and rs‐fMRI studies exemplifies the importance and potential scientific, social, and clinical value of conducting additional rs‐FC studies in GD individuals and comparing them with their aspired and assigned gender.

Given the inherent limitations of the aforementioned single‐case study (i.e. low statistical power, restricted to specific seed‐regions), the inconsistence of previous findings, and the potential scientific and clinical implications of future rs‐fMRI studies in GD, we aimed at investigating rs‐FC in a sample of hormonally untreated and treated MtFs in comparison with men and women. In our previous study, we found differences between MtFs and men or women in regions relevant to voice processing and cognitive demands (Junger et al., 2014). To examine if these findings are task‐specific or gender‐dependent, we chose to analyze those networks including the aforementioned regions (i.e. default mode, cerebellum, auditory, executive control, left and right fronto‐parietal, and medial visual). We hypothesized group differences in those networks to be present already in untreated MtFs. On a more general level, we set out to investigate whether brain connectivity in MtFs is more similar to their assigned gender, their aspired gender, or neither of those.

## 2 Methods and Materials

### 2.1 Participants

Twenty‐eight MtFs (15 hormonally untreated), 21 male and 20 female healthy control participants took part in this study. MtFs were recruited in self‐help groups, at the Department of Phoniatrics, Pedaudiology and Communication Disorders, and at the Department of Gynecological Endocrinology and Reproductive Medicine of the RWTH Aachen University Hospital. Hormonally treated MtFs have received treatment according to the German transsexual law (Schneider, Frister, & Olzen, 2010) for at least 3 months and had overcome the first phase of endocrinological adjustment. Untreated MtFs fulfilled diagnostic criteria for gender dysphoria and declared their intention of undergoing cross‐sex hormone therapy in the future. The German version of the Structured Clinical Interview of the fourth edition of the Diagnostic and Statistical Manual of Mental Disorders (DSM‐IV) (Wittchen, Zaudig, & Fydrich, 1997) was used to ensure the exclusion of participants with mental disorders unrelated to GD. Further exclusion criteria were neurological disorders, other medical conditions affecting the cerebral metabolism, and first degree relatives with a history of mental disorders. All participants were native German speakers and right‐handed except one left‐handed participant in each group. Handedness was assessed by means of the Edinburgh Handedness Inventory (Oldfield, 1971).

The hormonal status was obtained on the day of testing, except for three participants from whom no or only some blood parameters were available because of technical issues. Participants took part in two functional MR tasks, which are partly reported elsewhere (Junger et al., 2014). Four participants were excluded because of excessive movement in the scanner. Hence, data from 65 participants (14 untreated MtFs, 12 treated MtFs, 20 women, 19 men) were included in the final analyses. Groups did not differ significantly regarding age, years of education or crystallized verbal intelligence, but with respect to hormonal level of estradiol and testosterone (see Table 1). The number of hetero‐ and homosexual participants was equal in both MtF samples. (Sexual orientation in MtFs was defined according to their anatomical sex, i.e. homosexual MtFs prefer male partners; Table 1). The local Ethics Committee of the Medical Faculty of the RWTH Aachen University approved the study (reference: EK 088/09). Participants were financially reimbursed and gave their written informed consent.

**Table 1:** Characteristics of the sample (mean and standard deviations for age, years of education, IQ, and hormonal level) and group comparisons

	Men	Women	MtF untreated	MtF treated	*p* (ANOVA)
Age	32.32 (10.69)	32.50 (12.37)	35.50 (13.81)	32.42 (11.81)	.869
Education	14.58 (3.12)	14.85 (3.15)	14.50 (3.01)	13.83 (3.43)	.852
IQ	112.00 (12.34)	112.20 (15.71)	111.50 (13.09)	104.08 (6.56)	.310
Hormonal level					
17‐ß‐Estradiol (pmol/l)	90.51 (35.10)	131.89 (132.25)	87.16 (54.19)	663.28 (528.48)^a^	<.001*
Progesterone (nmol/l)	2.31 (1.10)	4.35 (8.46)	1.93 (0.77)	1.58 (0.80)	.355
Free testosterone (pmol/l)	37.12 (14.78)^b^	3.73 (2.27)^c^	36.42 (14.72)^b^	4.60 (6.22)^c^	<.001*

Significant differences are marked with asterisks.

a Significant difference with respect to all three other groups, Bonferroni corrected at *p *=* *.004.

b Significant differences with respect to women and MtF treated, Bonferroni corrected at *p *=* *.004.

c Significant differences with respect to men and MtF untreated, Bonferroni corrected at *p *=* *.004.

### 2.2 Data acquisition

Using a 3 Tesla Siemens Trio MR Scanner (Siemens Medical Systems, Erlangen, Germany) located at the Department of Psychiatry, Psychotherapy and Psychosomatics of the RWTH Aachen University Hospital, the following sequences covering the entire brain were obtained for each participant: (a) 4 min T1‐weighted MP‐RAGE 3D measurement (TR = 1900, TE = 2.52, TI = 900; α = 9°, FoV = 250 mm^2^, voxel size: 1 × 1 × 1 mm³, slices = 176); and (b) a 6.2 min T2*‐weighted echo‐planar imaging (EPI) resting‐state condition (TR = 3000, TE = 35, α = 84°, FoV = 192 mm, voxel size: 3 × 3 × 3 mm³, 44 slices, gap 15%, 64 × 64 matrix, repetitions = 124). For the resting‐state condition, participants were asked to relax in the scanner, keep their eyes open and avoid falling asleep.

### 2.3 VBM analysis

Previous studies have demonstrated that sex‐specific differences in brain morphometry can influence rs‐FC (Filippi et al., 2012). To account for those potential differences in the structural measures of the brain, voxel‐based morphometry (VBM) was used to account for the impact of potential sex‐specific differences in brain morphometry. Using the VBM8 toolbox implemented in SPM8 (http://www.fil.ion.ucl.ac.uk/spm) running within MATLAB 2010 (Mathworks, Sherborn, MA, USA), T1 images were normalized to template space and segmented into gray matter (GM), white matter (WM) and cerebrospinal fluid (CSF). Resulting GM maps were smoothed using an 8‐mm full‐with at half‐maximum Gaussian kernel, re‐sampled to equal voxel size (2 × 2 × 2 mm³) and image dimension (91 × 109 × 91) and included in the statistical analysis as a covariate.

### 2.4 Rs‐fMRI pre‐processing

Functional data were preprocessed using SPM8. Images were realigned to the mean image, co‐registered to the structural T1 image of each participant (which were segmented using ICBM template maps, aligned with atlas space, classified into GM, WM, and CSF and registered to MNI space), spatially normalized into MNI space, interpolated to 2 × 2 × 2 mm^3^ voxel size and smoothed with an 8 mm FWHM Gaussian kernel. A 110 Hz high‐pass filter removed effects of low‐frequency noise.

### 2.5 Probabilistic ICA and extraction of network components

Using the FSL Toolbox MELODIC (FMRIB, www.fmrib.ox.ac.uk/fsl/melodic2/index.html) a probabilistic independent component analysis (pICA) was performed. To avoid the magnetic field saturation effects, the first 3 functional images were discarded. The resulting 121 preprocessed functional images of each participant over time were concatenated into one 4D image. The ICA algorithm estimated the amount of noise and signal within the data and segmented it into spatially independent components each characterized by a consistent time course. This approach provides intensity z‐values for each voxel and its contribution to the time course of each component. Thus, individual components are the result of a multiple‐regression model enabling voxel‐wise quantitative measures of FC (Beckmann & Smith, 2004).

An in‐house MATLAB script, which was based on protocols of the previous studies (Clemens, Jung, et al., 2014; Clemens, Voss et al., 2014; Greicius, 2008; Greicius, Srivastava, Reiss, & Menon, 2004; Mingoia et al., 2012) was used to select those components optimally representing the individual functional networks. From the paper by Smith et al. (2009), we choose 7 different RSNs for our analyses: default mode, cerebellum, auditory, executive control, left and right fronto‐parietal, and medial visual. These RSNs were chosen because they all contained brain regions, which exhibited different neuronal activation between MtFs, men and women in our previous study (Junger et al., 2014). The script compared templates of the chosen functional networks, which were taken from Smith et al. (2009), with all components resulting from the pICA. Further, the script compared all components with inverse masks of the mentioned networks. Mean z‐values of both comparisons were extracted for all components. The difference between the mean z‐value for all voxels inside the template and the mean z‐value for all voxels outside the template was used to calculate a “goodness‐of‐fit”‐index for each participant and each component. The components with the best fit for each network were chosen. As a quality measure, we took the smallest “goodness‐of‐fit” index out of all components in each participant and calculated the mean and standard deviation for each group. Then, we excluded participants from the analyses for a specific RSN if the goodness‐of‐fit was smaller than the group mean minus the group standard deviation.

Only components above this cut‐off value were selected for further analyses. We compared this procedure for three pICA analyses which were set to output 21, 25, and 31 components, and we found the pICA yielding 25 components to provide the best results in terms of the highest total goodness‐of‐fit indices. Therefore, single‐subject components out of the 25 component pICA analysis were selected for further 2nd level analyses. The executive control and the cerebellar network were excluded from further analysis because of low goodness‐of‐fit indices in our sample, indicating that these RSNs could not be reliably detected in the current sample. Thus, all subsequent analyses were performed in the remaining 5 RSNs: default mode, auditory, left and right fronto‐parietal, and medial visual.

### 2.6 Whole sample analyses

In order to evaluate whether the selected components indeed represented the proposed RSN as previously defined by Smith et al. (2009) a whole group analysis of variance (ANOVA) for each network was performed using SPM8 and an *F* contrast (*p *< .05 FWE; extend threshold 80 voxel). The main purpose of this step of the analysis was to visually examine the degree of overlap between the RSN selected in the present study and the RSN templates obtained in previous studies.

### 2.7 Statistical group analyses

For the five RSN selected, three different general linear models (GLM) were calculated: containing either (1) two groups (untreated and treated MtFs); (2) four groups (men, women, untreated, and treated MtFs), or (3) three groups (men, women, and all MtFs pooled together). Based on previous studies incorporating structural measures into analyses of rs‐FC (e.g., Filippi et al., 2012), regional GM volumes were included as nuisance covariates using the Biological Parametric Mapping (BPM) toolbox (Casanova, Whitlow, Wagner, Espeland, & Maldjian, 2012). Resulting effects were compared between groups by means of two sample‐t tests, applying a family‐wise error (FWE) cluster level corrected statistical threshold of 0.05 (extent threshold 80 voxel).

## 3 Results

### 3.1 Resting‐state fMRI

**Figure 1:**
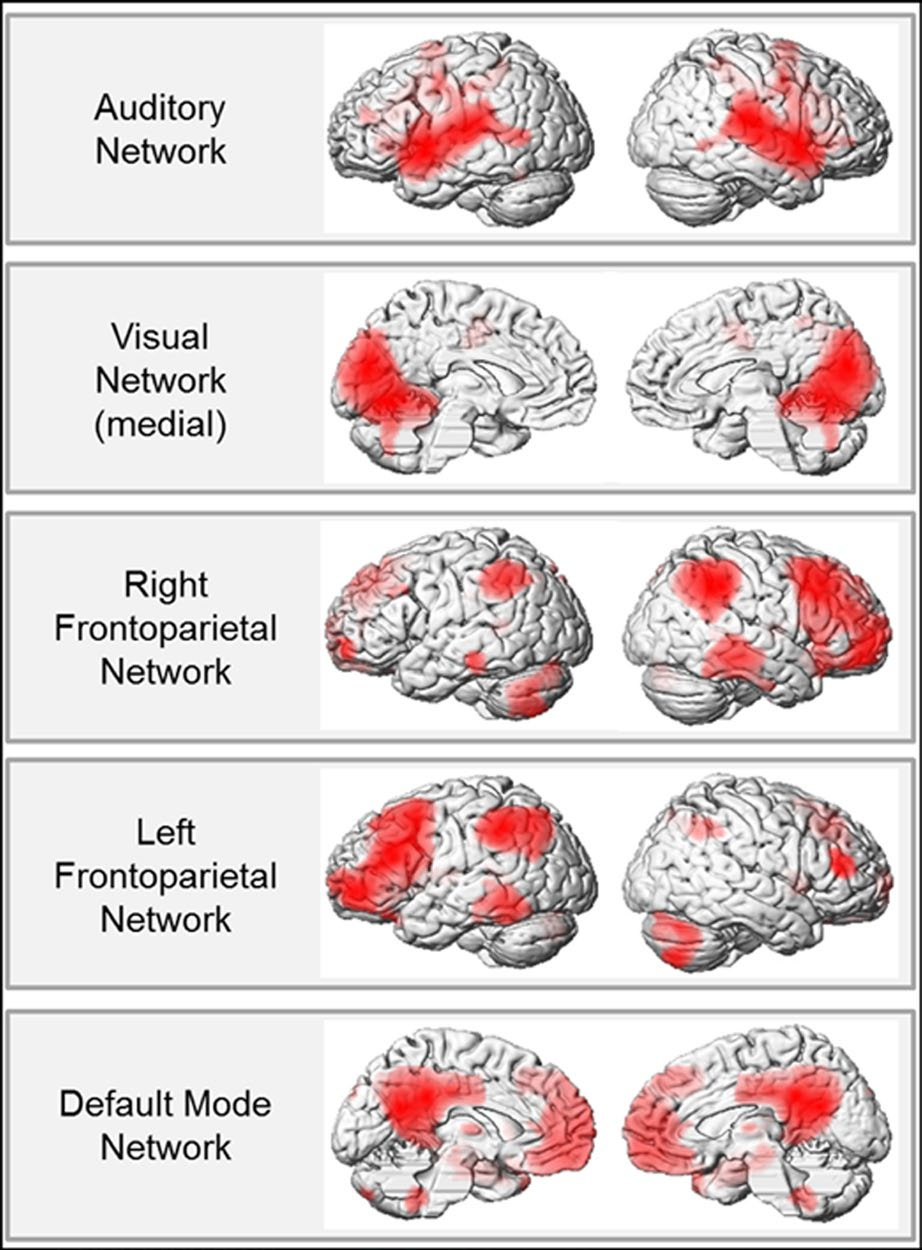
Functional network connectivity for the whole sample representing 5 different resting‐state networks (RSN) (*p *< .05 FWE cluster level corrected, extent threshold = 80 voxels). All RSN resemble and exhibit good overlap with the original RSN, which were taken from Smith et al. (2009)

One‐sample analyses of the pooled group revealed that the remaining five canonical resting‐state networks (default mode, auditory, left and right fronto‐parietal, and medial visual) were represented appropriately (Figure 1). Visual inspection revealed good anatomical overlap between the RSN selected here and the original RSN templates derived from Smith et al. (2009). After inspection of all contrasts of interest, no group differences were found for the medial visual and the default mode network. Thus, the following results describe differences, which were present in the auditory, left or right fronto‐parietal RSN.

### 3.2 Differences in network connectivity between control men and control women

When using the GLM containing three groups (men, women, and all MtFs pooled together), we found differences between control men and control women in the auditory network (Table 2). Compared with women, men revealed differential FC in left Heschl gyrus. There were no significant differences in FC between both control groups for the remaining networks.

**Table 2:** Connectivity group differences between men, women, untreated (UT) MtFs, treated (T) MtFs, or both MtF groups pooled together (MtF) regarding the functional resting‐state networks after correcting for GM volume (*p *< .05 FWE cluster level corrected, extent threshold >80 voxels; peaks MNI coordinates, *t* values [*t*] and cluster extensions [k])

Network	Contrast	Brain region	L/R	x	y	z	*t*	*k*
Auditory	Men > Women	Heschl Gyrus	L	−42	−26	10	3.90	143
	Men > MtFs (UT)	Inferior temporal gyrus	R	54	−16	−18	4.83	251
		Calcarine gyrus	R	16	−62	18	4.01	177
	MtFs (UT) > Women	Dorsomedial prefrontal cortex (DMPFC)	L	−6	32	50	4.68	172
		Midcingulate cortex	R	6	2	44	4.38	366
		Posterior cingulate cortex	L	−4	−20	28	4.59	177
		Inferior parietal gyrus	R	52	−26	28	4.14	151
	MtFs (T) > MtFs (UT)	Calcarine gyrus	R	8	−58	12	4.09	144
	MtFs (UT) > MtFs (T)	Thalamus	L	−10	−12	6	4.95	99
Right Fronto‐parietal	MtFs > Women	Thalamus	R	6	−22	2	4.64	178
Left Fronto‐parietal	Men > MtFs (T)	Dorsolateral prefrontal cortex (DLPFC)	L	−36	58	14	4.49	132
	MtFs (T) > MtFs (UT)	Inferior frontal gyrus (triangular part)	L	−42	34	2	4.67	137

### 3.3 Hormone treatment‐related differences in network connectivity within MtFs

**Figure 2:**
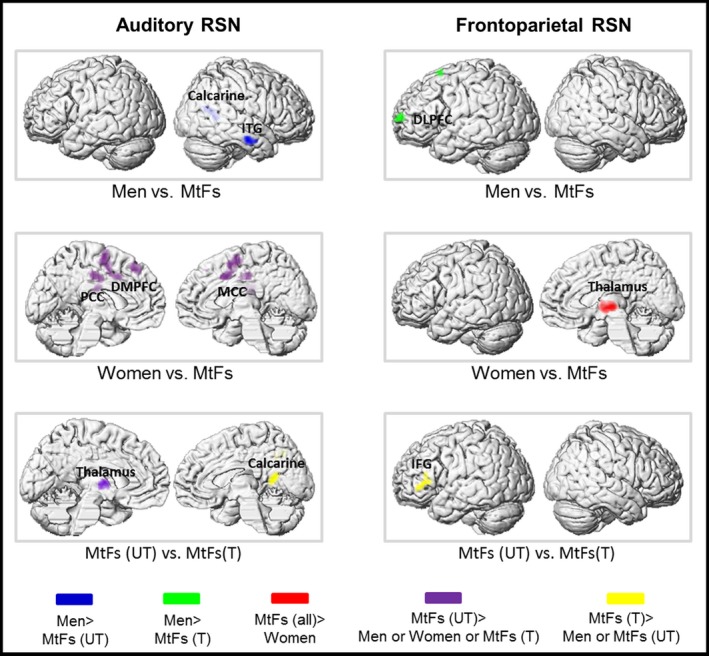
Group effects on resting‐state functional connectivity. Depending on whether differences between hormonally treated (T) and untreated (UT) MtFs in each network were found, results of the ANCOVAs are either presented for both MtF groups separately or pooled together and compared with men and women (*p *< .05 FWE cluster level corrected, extent threshold = 80 voxels). (DLPFC, dorsolateral prefrontal cortex; DMPFC, dorsomedial prefrontal cortex; IFG, inferior frontal gyrus; ITG, inferior temporal gyrus; MCC, middle cingulate cortex; PCC, posterior cingulate cortex)

Our analysis yielded differences between untreated and treated MtFs in the left fronto‐parietal and auditory network (Table 2; Figure 2). Untreated compared with treated MtFs showed stronger FC of the thalamus. Treated compared with untreated MtFs revealed increased FC in the calcarine gyrus and the interior frontal gyrus. There were no significant differences in FC between both MtF groups in the other networks.

### 3.4 Differences in network connectivity between men, women, and MtFs with or without hormonal treatment

On the basis of the observed differences between hormonally untreated and treated MtFs in the auditory and left fronto‐parietal network mentioned above, group differences in these networks were assessed (Table 2).

We found stronger FC in men of the inferior temporal and calcarine gyrus compared with untreated MtFs and in the dorsolateral prefrontal cortex compared with treated MtFs. In contrast, untreated MtFs revealed stronger FC of the dorsomedial prefrontal and the middle and posterior parts of the cingulate cortex as well as inferior parietal gyrus compared with women (Table 2; Figure 2).

### 3.5 Differences in network connectivity between control men, control women and all MtFs pooled together

When using the GLM containing three groups (men, women, and all MtFs pooled together), in the context of the right fronto‐parietal network, MtFs compared with women revealed stronger FC in the thalamus (Table 2; Figure 2). No significant differences were present in all other group comparisons.

## 4 Discussion

While little is known about the etiopathogenesis of gender dysphoria (GD) (Medras & Jozkow, 2010), functional neuroscience revealed evidence for greater similarities of GD individuals with their aspired than with their assigned gender in tasks differentiating between men and women (Carrillo et al., 2010; Gizewski et al., 2009; Schoning et al., 2010; Ye et al., 2011). Because of its stimulus‐unrelated and task‐free methodology, rs‐fMRI provides an unbiased strategy to investigate neurobiological functioning in GD and allows direct comparison of participants with their assigned and aspired gender. Previous studies already demonstrated gender‐related differences in FC (Filippi et al., 2012; Schoonheim et al., 2012). To the best of our knowledge, we provide the first rs‐FC study comparing both men and women with a group of MtFs. Interestingly, MtFs revealed patterns of rs‐FC that were different from both men and women: whereas rs‐FC in some networks was more similar between MtFs and women, as compared with MtFs and men, the opposite was the case for other networks. Thus, MtFs presented a unique pattern of rs‐FC, which cannot be easily assigned to either of the two sexes. This confirms previous findings suggesting an exceptional position of MtFs distinct from men and women (Luders et al., 2009).

### 4.1 Connectivity differences because of hormone therapy

The thalamus represents the relay between the inferior colliculus and the auditory cortex (Gruters & Groh, 2012) and with its extensive interconnectivity is known to transmit information of sense and consciousness to the frontal, temporal, and occipital lobes. Untreated MtFs revealed stronger FC as compared with treated MtFs in the thalamus in the context of the auditory network. Similarly, stronger FC of the thalamus was found in men compared with women (Tomasi, Chang, Caparelli, & Ernst, 2008). Thus, our findings indicate that MtFs before hormonal treatment resemble their assigned, and not their aspired gender. This corroborates the only previous rs‐FC study in GD, which revealed greater similarity of an FtM individual with female control participants (Santarnecchi et al., 2012).

However, this might change after hormonal treatment, as can be seen when examining the current findings regarding the inferior frontal gyrus (IFG). This region is strongly connected to the left amygdala in the context of self‐referenced positive inner speech and rumination as coping mechanism and this connectivity is higher in women compared with men (Kogler et al., 2016). Further, Witte et al. (2010) found thicker GM associated with higher estradiol levels and thinner GM with higher testosterone levels in left IFG. This is in line with our findings of stronger FC in treated MtFs compared with untreated MtFs in the IFG in the context of the left fronto‐parietal network. Concerning the thalamus and the IFG, untreated MtFs seem to resemble their assigned and treated MtFs their aspired gender, which might be related to differences in the hormonal level. Similar MtFs showed stronger FC than women in the thalamus in the context of the right fronto‐parietal network possibly driven by the untreated MtFs. Overall, this indicates a rather profound brain reorganization taking place during and after hormonal treatment in the GD individuals. Future research needs to clarify whether such changes in the brain connectivity correlate with behavioral and psychological changes often reported by GD individuals following hormonal treatment.

Furthermore, men as well as treated MtFs revealed stronger FC than untreated MtFs in the calcarine cortex. Animal studies comparing male and female rats have shown that similar testosterone levels in adolescence lead to different synaptic density in typical visual regions such as calcarine cortex, but end up in similar synaptic density in adulthood (Muñoz‐Cueto, García‐Segura, & Ruiz‐Marcos, 1990). In addition, Bramen et al. (2012) found high testosterone levels to be associated with thinner GM in girls and with thicker GM in boys. In line with these results, untreated MtFs seem to resemble adolescent girls, having similar testosterone levels to adult men, but lower connectivity. Hormonal treatment of MtFs seems to regulate the pattern such as in adult women which show the same pattern than men. This might explain why we found no difference in FC between men and women in this region.

### 4.2 Differences between men and MtFs

Differences between men and untreated MtFs were only found in the auditory network and resemble those in FC between men and women in the right inferior temporal gyrus (Schoonheim et al., 2012) and right calcarine gyrus (Biswal et al., 2010). Furthermore, the right inferior temporal gyrus revealed gender differences in morphometric connectivity (Gong, He, & Evans, 2011). Thus, connectivity differences in auditory processing seem to be at earlier processing stages such as primary visual cortex relevant for visual attention (von Kriegstein, Eger, Kleinschmidt, & Giraud, 2003) as well as in emotional (Habel, Klein, Kellermann, Shah, & Schneider, 2005) and semantic (Raettig & Kotz, 2008) word processing.

Furthermore, stronger FC in the left DLPFC as part of the fronto‐parietal network in men as compared with treated MtFs resemble evidence for a stronger FC in men compared with women in the context of other cognitive tasks (Kana, Murdaugh, Wolfe, & Kumar, 2012) as well as in rs‐fMRI (Koenig et al., 2013). This indicates that not only women but also treated MtFs reveal lower connectivity in cognition‐related areas as compared with men suggesting a different strategy in information processing (Piefke, Weiss, Markowitsch, & Fink, 2005). Thus, hormone treatment indeed seems to shift MtFs more toward their aspired gender, at least with respect to rs‐FC.

### 4.3 Differences between women and MtFs

Aside from the thalamus (discussed above) differences in FC to women were only present in untreated MtFs. This is in line with Ye et al. (2011), who found FC changes in direction to the aspired gender in GD individuals after cross‐sex hormonal treatment. Thus, although differences were found between untreated MtFs and women in the functional network connectivity, they were absent in hormonally treated MtFs.

Considering that MtFs identify with women, this is in line with evidence for estradiol enhancing right hemispheric functioning in women (Bayer & Hausmann, 2009; Hausmann, Becker, Gather, & Gunturkun, 2002; Weis et al., 2008) in regions involved in the fronto‐parietal network (Saletu et al., 2005). Even low doses of estradiol facilitate the functioning of brain regions involved in visual perception and attentional processes (Stevens, Clark, & Prestwood, 2005).

Increased FC between the auditory network and the dorsomedial prefrontal as well as middle and posterior cingulate cortex is associated with attention‐related emotional processing (Kim et al., 2012). In addition, both regions have been shown to be active in tasks paying attention to speech stimuli beyond auditory cortices (Husain et al., 2006). Furthermore, there is evidence for a greater activation in DMPF and cingulate cortex in men compared with women showing similar performance in response inhibition tasks (Li, Huang, Constable, & Sinha, 2006). This indicates a different strategy between men and women concerning attention‐related auditory processing observable in stronger FC between and greater activation of these regions in men. Concerning higher processing stages, untreated MtFs thus seem to resemble more their assigned gender.

### 4.4 Limitations

Due to the fact that the recruitment of GD individuals who (1) fulfill all inclusion criteria, (2) but none of the exclusion criteria, and (3) are willing to take part in our study was extremely difficult, we decided to include two left‐handed GD individuals as well. To equalize this aspect of the sample, we included one left‐handed subject in both male and female control groups as well. However, because of the fact that rs‐FC might differ between right‐handed and left‐handed subjects, we re‐ran all analyses excluding the left‐handers and observed similar findings without changes to the significant results. Thus, probably because of the low number of left‐handed subjects in our sample, we are convinced that the influence of handedness on the current results is marginal. Nevertheless, future studies should try to circumvent this limitation by either including only right‐handed subjects, or even better, by explicitly comparing rs‐FC between right‐handed and left‐handed GD individuals.

Another limitation of the present study relates to the fact that the acquisition of resting‐state data comprised only 124 volumes. This rather short resting‐state sequence was chosen because subjects had to complete two other fMRI tasks within the same MR session. However, it has been shown that the longer the resting‐state sequence, the better the measurement reliability, because the sampling variability decreases with increasing number of scans (Shehzad et al., 2009). Therefore, it has to be evaluated in future studies, whether comparable results can be obtained with longer resting‐state sequences.

## 5 Conclusion

Exploring FC via ICA, differences between sex groups were only found in auditory and fronto‐parietal RSNs. There were marked differences in FC between hormonally untreated and treated MtFs, indicating a strong influence of hormonal treatment. Differences to women were absent in treated MtFs, but present compared with untreated MtFs. In auditory processing untreated MtFs showed differences compared with women in earlier and compared with men in higher processing stages. In line with previous structural studies (Luders et al., 2009; Rametti et al., 2011) our data underline the exceptional position of MtFs. Hormonal treatment seems to shift MtFs more toward their aspired gender. Therefore, despite their biological sex MtFs revealed a distinct connectivity pattern especially different from those of men and partly also from those of women. Thus, the present study can be seen as a starting point, or pilot study, paving the way for future investigations designed to clarify whether rs‐FC in individuals with GD is indeed different from both their assigned and their aspired gender. What we can conclude already from the present study is that the hormonal treatment exerts a rather profound and strong effect on rs‐FC, indeed shifting patterns more toward the aspired gender. This might be particularly interesting also for GD individuals themselves, as it might help them to make crucial decisions regarding potential hormonal treatments and further surgical interventions.

## Conflicts of Interest

The authors reported no biomedical financial interests or potential conflicts of interest.
